# TGF-β induces phosphorylation of phosphatase and tensin homolog: implications for fibrosis of the trabecular meshwork tissue in glaucoma

**DOI:** 10.1038/s41598-017-00845-x

**Published:** 2017-04-11

**Authors:** Nikoleta Tellios, Jillian C. Belrose, Alexander C. Tokarewicz, Cindy Hutnik, Hong Liu, Andrew Leask, Michael Motolko, Miho Iijima, Sunil K. Parapuram

**Affiliations:** 1 Department of Ophthalmology, University of Western Ontario, London, ON N6A 4V2 Canada; 2 Pathology and Laboratory Medicine, University of Western Ontario, London, ON N6A 5C1 Canada; 3 Department of Anesthesia & Perioperative Medicine, University of Western Ontario, London, ON N6A 5A5 Canada; 4 Dentistry, Schulich School of Medicine and Dentistry, University of Western Ontario, London, ON N6A 5C1 Canada; 5 Lawson Health Research Institute, St. Joseph’s Health Care, London, ON N6A 4V2 Canada; 6 Department of Cell Biology, Johns Hopkins University School of Medicine, Baltimore, MD 21205 USA

## Abstract

Fundamental cell signaling mechanisms that regulate dynamic remodeling of the extracellular matrix (ECM) in mechanically loaded tissues are not yet clearly understood. Trabecular meshwork (TM) tissue in the eye is under constant mechanical stress and continuous remodeling of ECM is crucial to maintain normal aqueous humor drainage and intraocular pressure (IOP). However, excessive ECM remodeling can cause fibrosis of the TM as in primary open-angle glaucoma (POAG) patients, and is characterized by increased resistance to aqueous humor drainage, elevated IOP, optic nerve degeneration and blindness. Increased levels of active transforming growth factor-β2 (TGF-β2) in the aqueous humor is the main cause of fibrosis of TM in POAG patients. Herein, we report a novel finding that, in TM cells, TGF-β-induced increase in collagen expression is associated with phosphorylation of phosphatase and tensin homolog (PTEN) at residues Ser380/Thr382/383. Exogenous overexpression of a mutated form of PTEN with enhanced phosphatase activity prevented the TGF-β-induced collagen expression by TM cells. We propose that rapid alteration of PTEN activity through changes in its phosphorylation status could uniquely regulate the continuous remodeling of ECM in the normal TM. Modulating PTEN activity may have high therapeutic potential to alleviating the fibrosis of TM in POAG patients.

## Introduction

Dynamic remodeling of the extracellular matrix (ECM) is necessary for development, wound healing and maintenance of normal tissue homeostasis^1^. A breakdown in dynamic remodeling of the ECM can result in fibrosis which is characterized by excess deposition of ECM molecules that destroy the normal architecture of the tissue, leading to the impairment of organ function. It is estimated that loss of organ function due to fibrosis, as in diabetic nephropathy, pulmonary fibrosis and liver cirrhosis, contributes to one-third of natural deaths world-wide^2^. Currently there is no cure available for fibrotic diseases.

One of the factors that has a major impact on excess ECM deposition in many fibrotic diseases is transforming growth factor-beta (TGF-β)^3^. Thus, cell signaling mechanisms by which TGF-β induces excess ECM deposition are well studied^4^. Conversely, TGF-β in normal tissues has pleiotropic roles in the maintenance of tissue homeostasis^5^ and does not cause fibrosis. It is plausible that TGF-β-mediated signaling pathways that induce fibrosis are suppressed or balanced by TGF-β-induced increase in matrix metalloproteinase activity that degrades ECM^6–9^. Thus, further investigation are required to delineate fundamental regulatory feed-back signaling mechanisms that could prevent fibrotic actions of TGF-β without affecting its normal homeostatic roles in tissues. Such signaling mechanisms when identified may also serve as effective therapeutic targets to prevent disease-associated fibrosis.

TGF-β decreases the levels of Phosphatase and tensin homolog (PTEN) in several transformed cell lines, including HaCaT, PANC-1 and CAPAN-1, and also in primary glomerular mesangial cells^10–13^. Crucially, PTEN is capable of regulating TGF-β signaling as a co-factor for Smad2/3 phosphatase^14^ and is now considered a major regulator of ECM deposition^15^. Deletion of the *Pten* gene in dermal fibroblasts of mice induces excess collagen deposition/fibrosis *in vivo*, while *in vitro* overexpression of PTEN in dermal fibroblasts from scleroderma patients decreases collagen production, reversing the fibrotic phenotype^16^. Inhibition of PTEN activity in fibroblasts also increases collagen deposition^17^. Additionally, decrease in PTEN levels has been reported in many fibrotic diseases, including rheumatoid arthritis^18^ and pulmonary fibrosis^19^.

PTEN is a dual phosphatase and its major function is to dephosphorylate phosphatidylinositol 3,4,5-trisphosphate (PIP3) to phosphatidylinositol 4,5-bisphosphate^20^, thus inhibiting the PI3-kinase/AKT signaling pathway. PTEN is also known to dephosphorylate focal-adhesion kinase, and Src homology 2 domain containing transforming protein^21^. By regulating these signaling pathways, PTEN is able to modulate multiple cellular activities, including contractility, survival, apoptosis, migration and, cell-ECM interaction and signaling^21^. PTEN expression and activity is controlled by several mechanisms, including miRNAs, non-coding RNAs, phosphorylation, acetylation, oxidation, S-nitrosylation, and ubiquitylation^22^. These numerous and intricate modes of regulation of PTEN expression and activity indicate fundamental role of PTEN in regulating dynamic ECM remodeling in tissues.

Regular homeostatic ECM remodeling that occurs in most tissues^1, 23^ results in a rate of collagen turnover which is usually slow, with collagen half-life estimated to be 15 years in the skin and 117 years in the cartilage^24^. Conversely, under homeostatic conditions, accumulating evidence indicates that remodeling of ECM in the trabecular meshwork (TM) tissue is nearly continuous^25^. The TM, located at the irido-corneal angle, consists of TM cells and their porous extracellular matrix through which the aqueous humor, the clear fluid in the anterior segment of the eye, drains into the episcleral veins. Appropriate resistance to the drainage of aqueous humor through the TM is vital for homeostatic intraocular pressure (IOP). The TM tissue, thus occupies a high stress environment with fluctuations in mechanical and fluid shear forces^26, 27^. Continuous remodeling of ECM is crucial for appropriate resistance to aqueous humor drainage and maintenance of normal IOP. Indeed, it is expected that previously undescribed cell signaling mechanisms drive the dynamic and continuous ECM remodeling in the TM.

In contrast to the scenario in healthy eyes, fibrosis of the TM can result in primary open-angle glaucoma (POAG), the most common type of glaucoma which affects nearly 74% of the 70 million glaucoma patients worldwide^28^. Any increase in ECM deposition in the TM increases the resistance to aqueous humor drainage leading to an increase in IOP and is associated with optic nerve degeneration and blindness in POAG patients^29–32^. Lowering the IOP is the only medical strategy known to delay onset and slow progression of vision loss in glaucoma^33, 34^. One strategy to lower the IOP is to prevent the fibrosis of the TM; however, this has not yet been accomplished^35^.

Increased levels of active TGF-β2 in the aqueous humor of POAG patients^36^ are implicated in the fibrosis of the TM^37^. However, inhibiting TGF-β signaling is not a solution as TGF-β is also vital for maintaining the immune-privilege of the eye^38^. Moreover, TGF-β is present in the normal aqueous humor and TM cells both express and secrete this cytokine^36, 39–42^ and yet does not cause fibrosis of the TM. Indeed, induction of ECM deposition by TGF-β concurrently stimulates localized ECM digestion at invadosomes by increasing matrix metalloproteinase-2 activity in TM cells^8^. Thus, there is still a need to further delineate the elements in the TGF-β signaling pathway in normal and fibrotic TM, taking into account their potential to regulate the expeditious deposition or degradation of ECM in normal TM. While many of the mechanisms that drive ECM remodeling in TM are known^43^, there still could exist novel fundamental signaling mechanisms that enable pro-fibrotic TGF-β to expeditiously contribute to the continuous remodeling of ECM by TM cells.

In this study, we have investigated the modulation and regulation of PTEN levels/activity by TGF-β in human TM cells. We report that, in TM cells, TGF-β-induced increase in collagen expression is associated with the phosphorylation of PTEN at residues Ser380/Thr382/383. Phosphorylation of PTEN at these residues is known to suppress the activity of PTEN. Exogenous overexpression of mutant form of PTEN with enhanced phosphatase activity prevented TGF-β-induced collagen expression by TM cells. We propose rapid alteration of PTEN activity through changes in its phosphorylation status to be a unique mechanism that regulates the continuous remodeling of ECM in the normal TM. PTEN or elements in its signaling pathway could serve as a therapeutic target with high potential to alleviate fibrosis of the TM in glaucoma.

## Results

### TGF-β induces an increase in levels of PTEN and its phosphorylation

**Figure 1:**
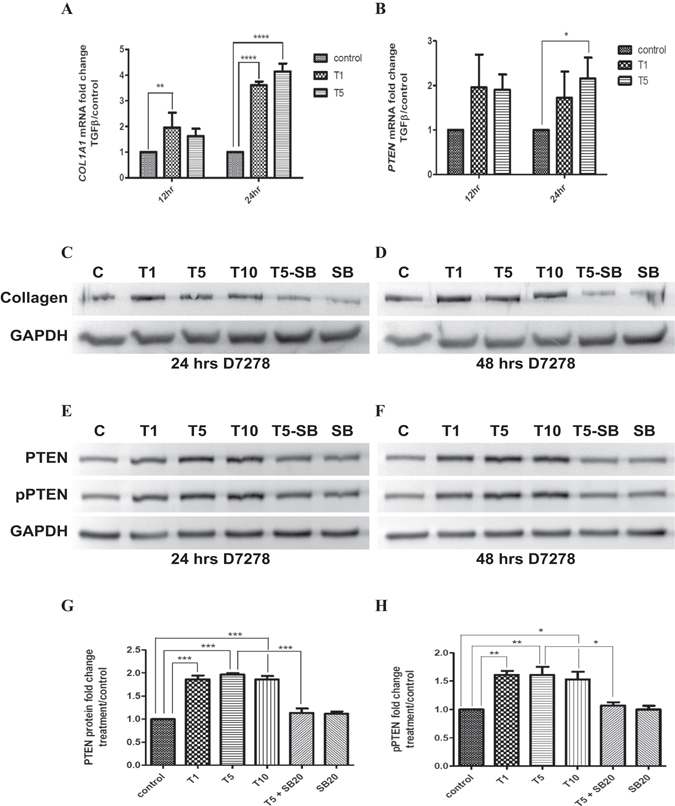
TGF-β induces an increase in levels of PTEN and its phosphorylation in human TM cells. (**A,B**) Real-time PCR analysis of *COL1A1* and *PTEN* mRNA levels in TM cells treated with TGF-β2 (1 or 5 ng/ml). RNA was extracted at 12 and 24 h. mRNA expression levels were normalized to EUK18S. A two-way ANOVA with Bonferroni’s post-hoc was used to determine statistical significance. *p < 0.05, **p < 0.01, ****p < 0.0001. N = 3. (**C,D**) Western blot analysis of collagen levels in TM cells treated with TGF-β (T1, T5, T10 indicates 1, 5 or 10 ng/ml TGF-β2). Protein was extracted at 24 and 48 h. TGF-β receptor signaling was inhibited using the inhibitor SB431542 (SB; 20 μM). D7278- representative human donor. (**E,F**) Western blot analysis of PTEN levels and its phosphorylation (pPTEN- ser380/thr382/383) in TM cells treated with TGF-β and SB431542. Protein was extracted at 24 and 48 h. D7278- representative human donor. (**G,H**) Densitometry was performed on PTEN and pPTEN Western blot data at 48 h using ImageJ software. Statistical significance was determined using one-way ANOVA with Tukey’s post-hoc. *p < 0.05, **p < 0.01, ***p < 0.001. N = 3. (I,J). TGF-β-mediated induction of PTEN levels and its phosphorylation was confirmed in TM cells from an adult human donor, aged 26 yrs (Adult 26Y).

Levels of TGF-β2 in the aqueous humor range from ~0.5 ng/ml to ~8 ng/ml^36, 42^, whereas glaucomatous aqueous humor have higher levels of total as well as active TGF-β2^36^. To explore the effect of TGF-β on ECM deposition, we treated TM cells with 1 ng/ml, 5 ng/ml, or 10 ng/ml of TGF-β2 to simulate physiological and pathological levels of TGF-β2 in the aqueous humor. Since TGF-β is a major inducer of fibrosis, addition of TGF-β to cultured human embryonic TM cells, as expected, increased collagen mRNA levels **(**Fig. 1A). Increase in COL1A1 mRNA is seen at 12 h, rising further to a 3.6 and 4.1 fold increase in mRNA levels at 24 h after 1 ng/ml and 5 ng/ml TGF-β treatment, respectively. A corresponding increase in collagen 1 protein levels are also seen in TM cells treated with TGF-β **(**Fig. 1C,D **)**. Surprisingly, TGF-β also significantly increased the levels of PTEN mRNA in TM cells, with a significant 2.2 fold increase after treatment with 5 ng/ml TGF-β for 24 h (Fig. 1B **)**. There is also an increase in PTEN protein levels in TM cells following TGF-β treatment for 24 h and 48 h; a nearly two fold increase is seen after 48 h of TGF-β treatment (Fig. 1E–G). However, the increase in PTEN protein levels was also accompanied by a concomitant increase in phosphorylation of residues Ser380/Thr382/383 in the PTEN tail region, with almost a 1.6 fold increase at 48 h (Fig. 1E,F,H). Phosphorylation of PTEN at these residues is indicative of inactivation of PTEN^44–48^. The increase in levels/phosphorylation of PTEN after TGF-β treatment was reproduced when TM cells were cultured on either tissue culture plastic or on collagen/pronectin-coated elastomer plates (data not shown).

### Association of PI3-kinase and PTEN in TGF-β-mediated collagen expression

**Figure 2:**
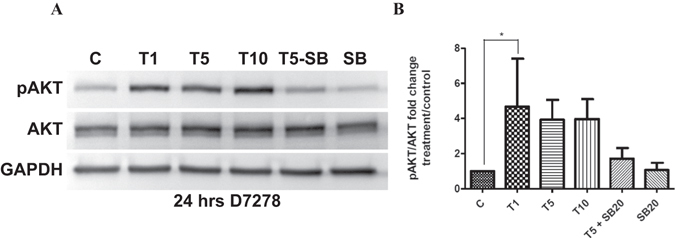
TGF-β induces phosphorylation of AKT in human TM cells. (**A**) Western blot analysis of AKT and its phosphorylation (pAKT- ser473) in TM cells treated with TGF-β2 (1, 5 or 10 ng/ml). Protein was extracted at 24 h. TGF-β receptor signaling was inhibited using SB431542 (SB; 20 μM). D7278- representative human donor. (**B**) Densitometry was performed on AKT and pAKT Western blot data using ImageJ software. Statistical significance was determined using one-way ANOVA with Tukey’s post-hoc. *p < 0.05. N = 3.

**Figure 3:**
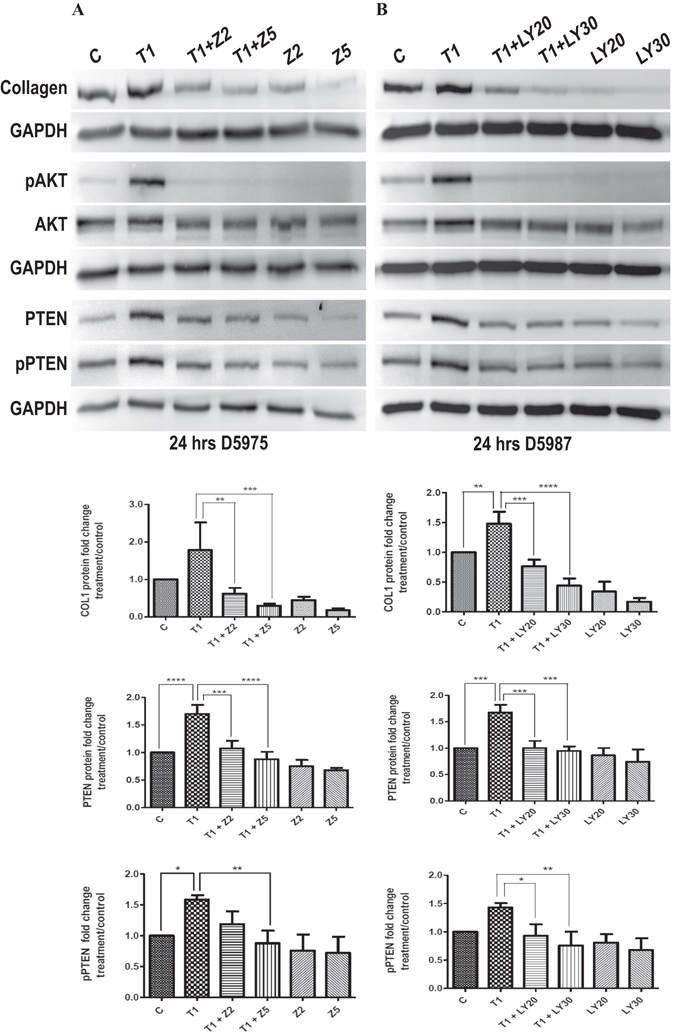
Inhibition of PI3-kinase/AKT signaling prevents TGF-β-induced increase in levels of collagen and PTEN/phosphorylation in human TM cells. (**A,B**) Human TM cells were treated with TGF-β2 (1 ng/ml) and PI3-kinase signaling inhibited using ZSTK474 (Z2, Z5- 2 μM or 5 μM) or LY294002 (LY20, LY30- 20 μM or 30 μM). Protein was extracted at 24 h and collagen, PTEN, pPTEN (ser380/thr382/383), AKT and pAKT detected by Western blot. Densitometry was performed on collagen, PTEN and pPTEN Western blots using ImageJ software and shown as graphs. Statistical significance was determined using one-way ANOVA with Tukey’s post-hoc. *p < 0.05, **p < 0.01, ***p < 0.001, ****p < 0.0001. N = 3. D5975, D5987- representative human donors.

Non-canonical TGF-β type I receptor (ALK5)/Smad-independent signaling is associated with enhanced collagen deposition^49–52^. In our experiments, we assessed the PI3-kinase pathway not only because active PTEN prevents the PI3-kinase-mediated AKT signaling, but also because PI3-kinase is able to block PTEN activity^53, 54^. We found that addition of TGF-β induced the phosphorylation of AKT (Ser473) in TM cells, indicating again that the phosphorylation of PTEN following TGF-β treatment has inactivated its phosphatase activity. A 4.7 fold increase in phosphorylation of AKT is observed following 1 ng/ml TGF-β treatment for 24 h (Fig. 2A,B). Inhibition of PI3-kinase signaling by ZSTK474 and LY294002 almost completely eliminated the TGF-β-induced phosphorylation of AKT. Both PI3-kinase signaling inhibitors also significantly suppressed TGF-β-induced collagen expression. We found a corresponding attenuation of PTEN and its phosphorylation at residues Ser380/Thr382/383 to levels similar to that of controls (Fig. 3A,B).

### Inhibition of PTEN and overexpression of PTEN in TM cells modulates collagen expression

**Figure 4:**
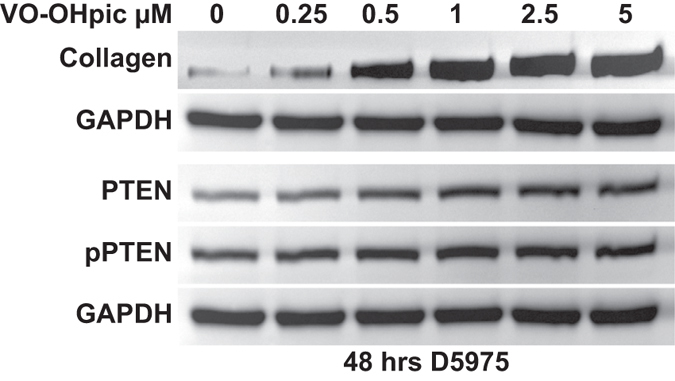
Inhibition of PTEN increases levels of collagen in human TM cells. Human TM cells were treated with VO-OHpic, a potent inhibitor of PTEN at varying concentrations. VO-OHpic at 5 μM caused a nearly 4-fold (N = 3) increase in collagen levels at 48 h. A corresponding increase in the levels of PTEN and phosphorylated PTEN was noted. D5975- representative human donor.

**Figure 5:**
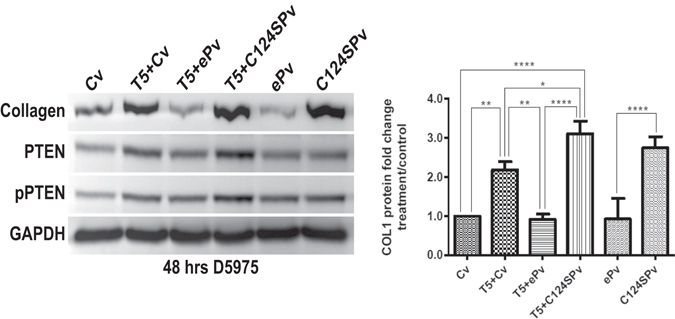
PTEN activity modulates collagen expression in human TM cells. TM cells were treated with TGF-β2 (5 ng/ml) and transfected with control GFP-vector (Cv), enhanced PTEN-GFP vector (ePv) or C124SPTEN-GFP vector (C124SPv). Enhanced PTEN has eight-fold increased ability to suppress PIP3 signaling, while C124SPTEN has defective lipid phosphatase activity. Densitometry was performed on collagen Western blots using ImageJ software. Statistical significance was determined using one-way ANOVA with Tukey’s post-hoc. *p < 0.05, **p < 0.01, ****p < 0.0001. N = 3. D5975- representative human donor. Transfection with enhanced PTEN decreased the levels of TGF-β-induced collagen to that of controls, while reducing the levels of PTEN and its phosphorylation. Transfection with C124SPTEN enhanced TGF-β induced increase in collagen levels and this was accompanied by an increase in the levels of PTEN and phosphorylated PTEN.

Since our results are consistent with the hypothesis that TGF-β regulates collagen deposition by modulating PTEN/AKT levels and phosphorylation, we further investigated whether inhibition of PTEN activity and its overexpression affected collagen expression by TM cells. PTEN activity was inhibited using VO-OHpic^55^; in TM cells application of 5 µM VO-OHpic increased the expression of collagen by nearly 4 fold after 48 h of treatment. VO-OHpic-induced increase in collagen levels was also accompanied by an increase in PTEN levels and its phosphorylation (Fig. 4). This further reinforces that changes in levels of PTEN/phosphorylation affects collagen expression. We also assessed the effect of mutant PTEN on collagen expression by transfecting TM cells with enhanced PTEN (ePTEN) which has an approximately eightfold increased ability to suppress PIP3 signaling, and by also transfecting PTEN with defective lipid phosphatase activity (C124SPTEN)^56^. Overexpression of ePTEN completely prevented TGF-β mediated upregulation of collagen expression by TM cells while decreasing PTEN/phosphorylation to levels similar to that of controls. Overexpression of C124SPTEN enhanced TGF-β-induced collagen expression by nearly 1.5 fold and enhanced the levels of PTEN/phosphorylation (Fig. 5). Preliminary experiments also indicate that overexpression of ePTEN and C124SPTEN has similar effects on the induction of fibronectin by TGF-β (Supplementary data Fig. 1); regulation of fibronectin expression by PTEN has been previously reported^57^.

## Discussion

TGF-β is a well-established inducer of collagen deposition by activation of canonical Smads and through non-canonical PI3-kinase and p38 MAPK signaling pathways^49–52, 58, 59^. However, the regulation of PTEN by TGF-β^10, 11, 13, 60^ and the increasingly key role of PTEN in the modulation of ECM deposition are recent findings^14, 17, 61, 62^. The role of PTEN in TGF-β signaling becomes crucial as it regulates TGF-β-induced collagen deposition by modulating Smad signaling^14^. Nuclear PTEN protects protein phosphatase, Mg^2+^/Mn^2+^ dependent 1 A (PPM1A, a Smad2/3 phosphatase) from degradation induced by TGF-β signaling. Any decrease in PTEN is thought to destabilize PPM1A, allowing Smad2/3 to remain phosphorylated in the nucleus, thus promoting TGF-β signaling^14^. We chose collagen I as the ECM protein to investigate as it is present in the striated collagen fibrils of the trabecular core, the basement membrane of the trabecular beams and in the loose aggregates in the juxtacanalicular tissue^63^ and is expressed by TM cells in culture^64^. Collagen I expression is also induced by TGF-β in TM cells^65^ and its accumulation causes increased IOP and progressive loss of optic nerve axons^66, 67^.

In this study we report that TGF-β treatment of TM cells induced expression of collagen despite an increase in the levels of PTEN. This is contrary to previous reports that an increase in the levels of PTEN reduced collagen deposition^17, 61, 68^. Our data suggest that TGF-β induced the expression of collagen by TM cells because the increase in PTEN levels is antagonized by an increase in its phosphorylation at Ser380/Thr382/383 in the C-terminal tail region. Phosphorylation at these residues is known to reduce PTEN activity^48^ and causes folding of the C-terminal tail on to the membrane-binding region, thus preventing translocation of PTEN to cell membrane, suppressing its enzymatic activity^44–48^. The inactivation of PTEN due to phosphorylation and its association with increase in collagen expression by TM cells is further supported by previous reports that *Pten* gene deletion or PTEN inhibition increased deposition of collagen^17, 61^.

The continuous remodeling of ECM in the TM reflects its adaptation to a high-stress mechanical stress environment and the necessity to maintain normal IOP, despite the persistent fluctuations of IOP^26^. Expeditious activation and deactivation of PTEN through alteration of phosphorylation status could be one of the fundamental signaling mechanisms that allow for continuous remodeling of ECM in TM tissue. Our study demonstrates for the first time that treatment with TGF-β is associated with phosphorylation of PTEN. Hitherto, TGF-β has only been known to decrease PTEN levels^10–13, 60^ in experiments mostly on transformed cell lines, representing tissues that do not need continuous ECM remodeling.

Reversible control of phosphorylation allows cells to respond rapidly to fluctuating changes in its environment^69^. Other factors present in the aqueous humor could balance the TGF-β-induced phosphorylation of PTEN. For example, Hepatocyte growth factor (HGF) is present in the normal aqueous humor^70^. HGF is known to inhibit TGF-β-induced decrease in PTEN levels^71^ and also antagonize the fibrotic actions of TGF-β^72^. Alternative isoform of TGF-β receptor II in TM cells could also explain the difference in TGF-β signaling mechanism in TM cells^73^. In this context, it is noteworthy that, in TM cells, TGF-β not only increases ECM deposition, but concurrently induces localized ECM digestion at invadosomes by increasing matrix metalloproteinase-2 activity^8^.

The increase in PTEN levels at 24 and 48 hours that we found following TGF-β treatment, could represent a feed-back mechanism to compensate for an early decrease in PTEN activity due to phosphorylation. Our preliminary experiments (Supplementary data Fig. 2) indicate an early increase in PTEN phosphorylation at 15 to 30 minutes after TGF-β treatment. This increase appears to attenuate at one hour after TGF-β treatment. The early increase in PTEN phosphorylation coincides with known peak phosphorylation of Smad3 at 30 minutes after TGF-β treatment^74, 75^, and is relevant as PTEN is known to regulate Smad signaling through Smad2/3 phosphatase PPM1A^14^. Moreover, being a highly regulated protein^22^, activation and recruitment to the membrane is known to rapidly degrade PTEN^44^, while phosphorylation at Ser380/Thr382/Thr383 increases its stability^48^. Thus, the increase in PTEN levels could also be a mechanism that promptly compensates for any activity-induced degradation of PTEN.

We further showed that, in TM cells, the increase in collagen after TGF-β treatment is associated with an increase in phosphorylation of AKT at Ser473. Since PTEN inhibits the PI3-kinase/AKT signaling pathway^76^, the increase in pAKT yet again indicates a reduction in PTEN activity. Conversely, we demonstrate that inhibition of the PI3-kinase pathway resulted in almost complete elimination of AKT phosphorylation by TGF-β, and also prevented TGF-β-induced increase in collagen levels. The decrease in AKT phosphorylation and collagen expression following inhibition of PI3-kinase pathway indicates an increase in PTEN activity. Activation of PTEN with inhibition of PI3-kinase pathway has been previously reported^53, 77, 78^. Moreover, inhibition of PI3-kinase pathway reduced TGF-β-induced increase in PTEN levels and phosphorylation. This again indicates a rapid degradation of PTEN following its activation and recruitment to the membrane^44^.

Additionally, we show that PTEN inhibition increased collagen production by TM cells. Furthermore, overexpression of ePTEN, with approximately eight-fold increased ability to suppress PIP3 signaling^56^, prevented TGF-β-induced collagen and fibronectin expression. In contrast, overexpression of PTEN with defective lipid phosphatase activity^56^ further enhanced TGF-β-induced collagen and fibronectin expression in TM cells. Considering that even a 20% reduction in PTEN levels induces early lethality and cancer susceptibility^79^, it is highly plausible that even a modest therapeutic increase in PTEN activity could effectively reduce fibrosis of TM in glaucoma.

New therapeutic approaches are critical, as progressive vision loss is common among glaucoma patients despite effective current treatments^80^. However, no new drug classes have been approved worldwide to treat glaucoma for nearly two decades^81, 82^. Several new drugs are currently in clinical trials but they have varying efficacies and side-effect profiles and strategies of treatment are still evolving^81, 82^. Our results indicate that PTEN has high potential to emerge as an effective therapeutic treatment for glaucoma. Several therapeutic drugs that indirectly enhance PTEN activity are currently available^83^, and may be used to prevent PTEN phosphorylation, increase PTEN activity or modulate elements associated with its signaling to alleviate fibrosis of TM.

## Methods

### Cell culture

For all experiments human trabecular meshwork cells at passage 4 were used. Donor cells 7278, 5975, and 5987 were of embryonic origin (ScienCell Research Laboratories, Carlsbad, CA). Phenotype of donor cells was further confirmed by immunostaining for von-Willebrand factor (data not shown) and by detection of myocilin after dexamethasone treatment (Supplementary data Fig. 3). Cells were cultured in low glucose DMEM (Life Technologies, Burlington, ON, Canada) supplemented with 10% FBS with 1% Penicillin-Streptomycin (Life Technologies) on either tissue culture-treated plastic or on Bioflex pronectin/collagen-coated (Flexcell International Corporation, Burlington, NC) plates. Cells were in low serum medium (0.5% FBS) for at least 6–12 h and nearly confluent when treated with human recombinant TGF-β2 (R&D Systems, Inc, Minneapolis, MN). When small molecule inhibitors of cell signaling were used [SB431542, VO-OHpic (Tocris Bioscience, Minneapolis, MN), LY294002, or ZSTK474 (Selleck Chemicals, Houston, TX)] the cells were pretreated for 30 min to 1 h.

### Real-time PCR

Total RNA was isolated using the RNeasy RNA extraction kit (Qiagen, Mississauga, ON) and quantity and integrity of RNA sample was checked using a Nanodrop 2000 (Thermo Scientific, Waltham, MA). 25 ng of total RNA was reverse-transcribed and amplified using one-step RT-qPCR master mix (qScript XLT One-step RT-qPCR ToughMix, ROX; Quanta Biosciences, Beverly, MA) and detected with ABI Prism 7900HT (Applied Biosystems/Thermo Scientific). TaqMan Assay-on-Demand primers (Applied Biosystems/Thermo Scientific) were used and samples were run in triplicate. Levels of *PTEN* and *COL1A1* mRNA expression in samples were normalized to values of control 18S and fold change determined using the 2^−ΔΔCt^ method.

### Western blots

Since increase in PTEN expression was surprisingly accompanied by an increase in its phosphorylation, Western blots to probe the expression of PTEN and phosphorylated PTEN were initially conducted separately (Supplementary data Fig. 4). This was to avoid any technical problem associated with stripping and reprobing the blots. Once the concomitant increase in both PTEN and phosphorylated PTEN was established, further experiments probed the same blot, first for phosphorylated PTEN and then followed by PTEN. Following detection of phosphorylated PTEN, blots were stripped and checked for any residual HRP-conjugated secondary antibody before being probed for PTEN.

Proteins were extracted using IP lysis buffer (Pierce; Thermo Scientific) and protein estimation was performed using the Micro BCA Protein Assay kit (Thermo Scientific). Proteins were resolved on polyacrylamide gels and blotted on to nitrocellulose membranes and probed with anti-PTEN rabbit pAb (AF847) (R&D Systems, Inc), anti-phosphoPTEN Ser380/Thr382/383 rabbit mAb (44A7) (Cell Signaling Technologies, Danvers, MA), anti-Akt (pan) rabbit mAb (C67E7) (Cell Signaling Technologies), anti-phosphoAkt Ser473 (D9E XP) rabbit mAb (Cell Signaling Technologies), anti-collagen type 1 rabbit mAb (EPR7785) (Abcam, Cambridge, UK) and anti-GAPDH mouse mAb (9B3: sc-66163) (Santa Cruz Biotechnology, Santa Cruz, CA). HRP conjugated anti-mouse and anti-rabbit antibodies were used in conjunction with an ECL imaging system. Densitometric analysis of western blot bands was performed using ImageJ software.

### Overexpression of PTEN

TM cells were transfected using the Polymag Neo Magnetofection Kit (OZ Biosciences Inc, San Diego, CA) according to manufacturer’s instructions. TM cells were transfected with 1 µg of either IRES GFP^84^ (Addgene, Cambridge, USA), ePTEN-GFP or C124SPTEN-GFP^56^ in pcDNA3.1 backbone, followed by addition of 5 ng/ml TGF-β2, 20–24 h after transfection. Protein was extracted at 24 and 48 h after addition of TGF-β to transfected TM cells.

### Statistical analysis

Two-way ANOVA with Bonferroni’s *post-hoc* test or one-way ANOVA with Tukey’s *post hoc* test was applied to determine the statistical significance in differences between treatment groups. Differences were considered statistically significant when P < 0.05. GraphPad Prism software (version 6.0, GraphPad Software, San Diego, CA) was used for all statistical analyses.

## Electronic supplementary material

Supplemental Figures
