# Supplementary material for: TGF-β induces phosphorylation of phosphatase and tensin homolog: implications for fibrosis of the trabecular meshwork tissue in glaucoma
Source: Sci Rep. 2017 Apr 11;7:812. doi: 10.1038/s41598-017-00845-x (PMC5429747; doi:10.1038/s41598-017-00845-x)
Supplement: Supplementary file 1 — Supplemental Figures [file 41598_2017_845_MOESM1_ESM.pdf]

TGF-β induces phosphorylation of phosphatase and tensin homolog: implications for fibrosis of the trabecular meshwork tissue in glaucoma

Nikoleta Tellios, Jillian C. Belrose, Alexander C. Tokarewicz, Cindy Hutnik, Hong Liu, Andrew Leask, Michael Motolko, Miho Iijima, Sunil K. Parapuram

Supplementary data Figure 1: PTEN activity modulates fibronectin expression in human TM cells. TM cells were treated with TGF-β2 (5 ng/ml) and transfected with control GFP-vector (Cv), enhanced PTEN-GFP vector (ePv) or C124SPTEN-GFP vector (C124SPv). Enhanced PTEN has approximately eight-fold increased ability to suppress PIP3 signaling, while C124SPTEN has defective lipid phosphatase activity. Preliminary experiments indicate that transfection with enhanced PTEN decreased the levels of TGF-β-induced fibronectin protein expression. Transfection with C124SPTEN enhanced TGF-β induced increase in fibronectin protein expression. The protein extracts from this experiment was also used to analyze collagen expression and the changes in collagen expression were similar to that described in Figure 5, Results section. D7278- human donor.

Supplementary data Figure 2: TGF-β induces an early increase in PTEN phosphorylation in human TM cells. Preliminary experiments indicate an increase in PTEN phosphorylation as early as 15 minutes in donor D5975 and at about 30 minutes in donor D5987, following treatment with 5ng/ml TGF-β2. This early increase in PTEN phosphorylation attenuates by one hour.

Supplementary data Figure 3: Characterization of TM cells. Dexamethasone induced the expression of myocilin in the three donor TM cells used in the experiments, indicating that majority of the cells had the phenotype of TM cells. Once the TM cells were confluent the medium was changed to low-serum for 24 h, followed by treatment with dexamethasone (500nM) for 48 h. Protein was extracted and myocilin detected by Western blot. D7278, D5975, D5987- human donors.

Supplementary data Figure 4: Since increase in PTEN expression was surprisingly accompanied by an increase in its phosphorylation, Western blots were initially conducted separately to confirm such an increase in expression of PTEN and phosphorylated PTEN. Here we show a technical replicate of a separate blot probed for PTEN using the same donor (D7278) as in Figure 1E. Phosphorylated PTEN image is same as in Figure 1E.

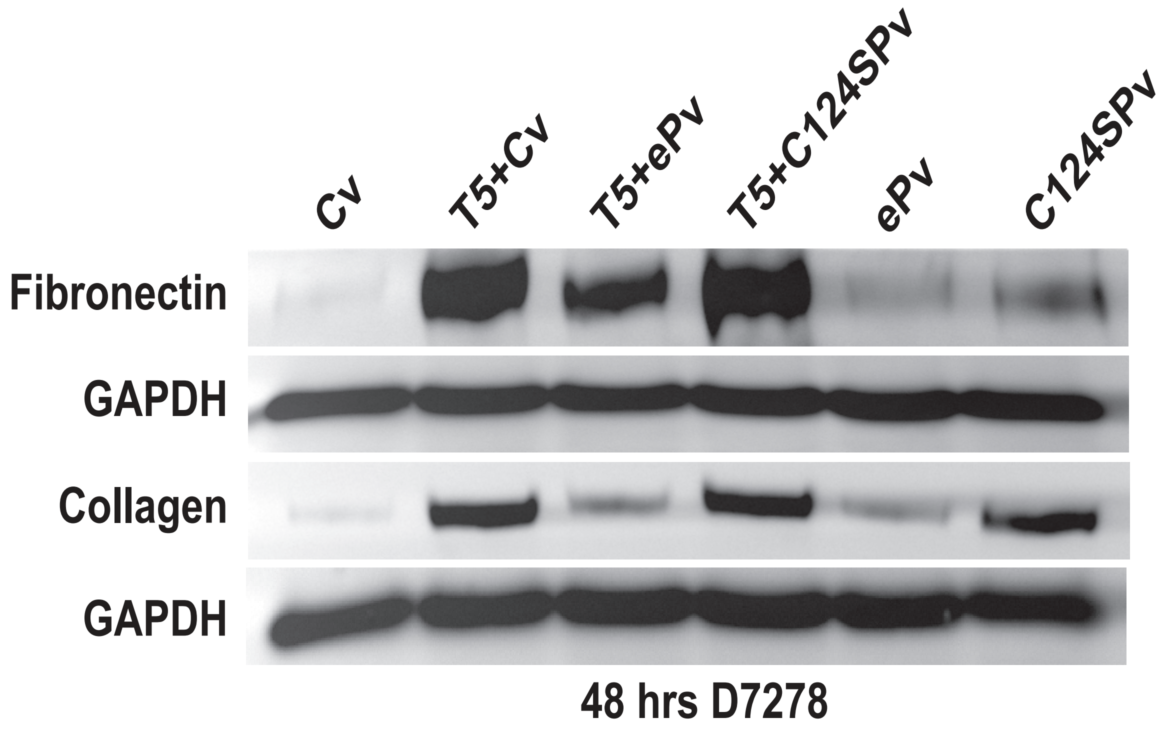
Cv
T5+Cv
T5+ePv
T5+C124SPv
ePv
C124SPv
Fibronectin
GAPDH
Collagen
GAPDH
48 hrs D7278

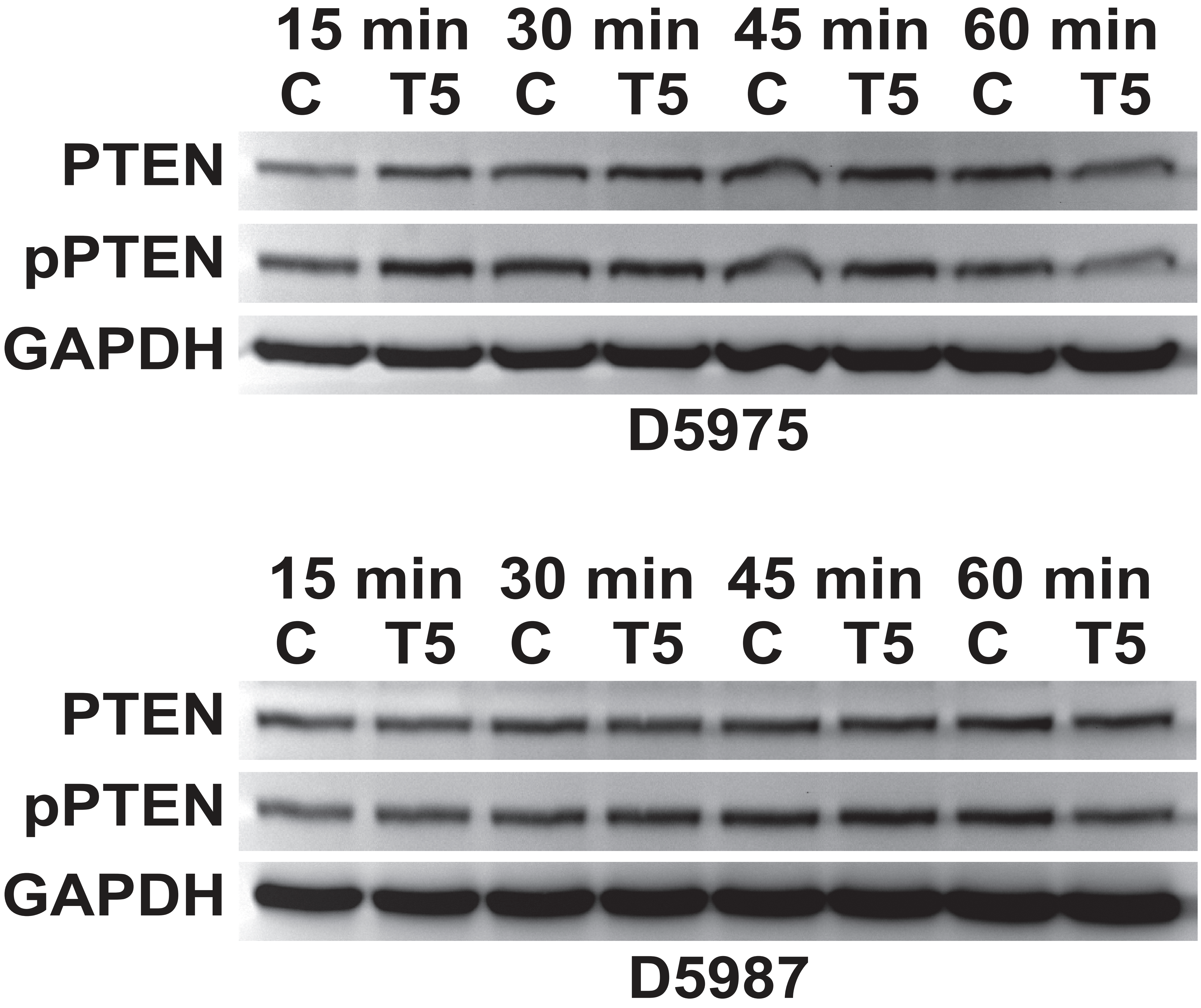
15 min 30 min 45 min 60 min
C T5 C T5 C T5 C T5
PTEN
pPTEN
GAPDH
D5975
15 min 30 min 45 min 60 min
C T5 C T5 C T5 C T5
PTEN
pPTEN
GAPDH
D5987

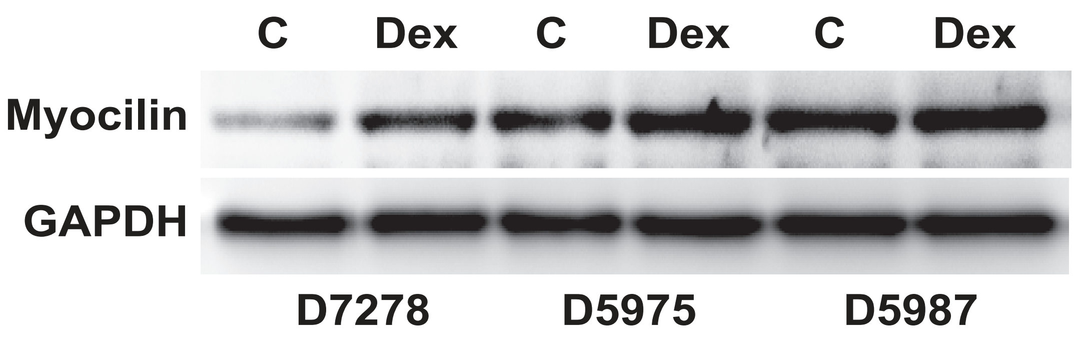
C
Dex
C
Dex
C
Dex
Myocilin
GAPDH
D7278
D5975
D5987

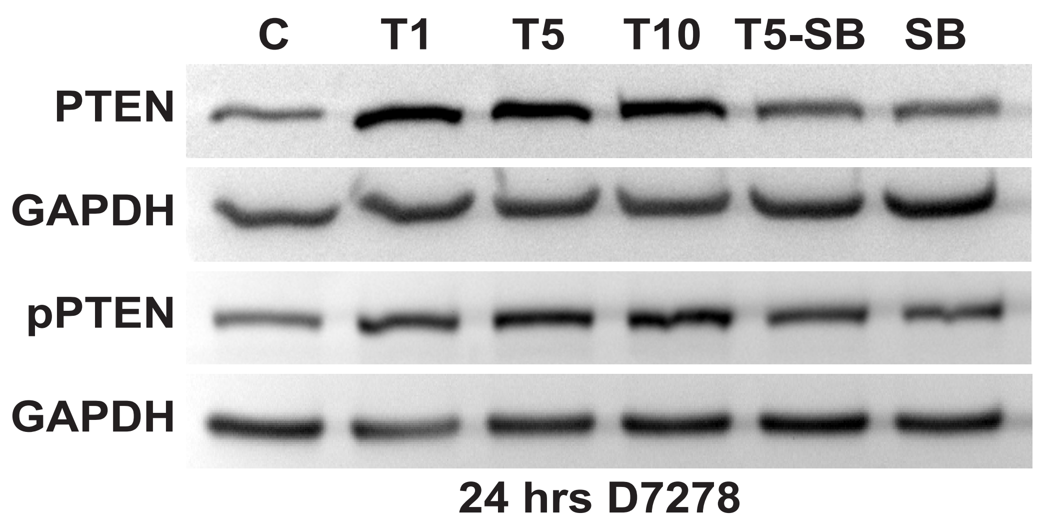
C
T1
T5
T10
T5-SB
SB
PTEN
GAPDH
pPTEN
GAPDH
24 hrs D7278
